# Long-term prognostic significance of ascites cytology in ovarian cancer cases in which R0 resection was achieved in the initial surgery: a multi-institutional retrospective cohort study

**DOI:** 10.1093/jjco/hyaf046

**Published:** 2025-03-20

**Authors:** Shohei Iyoshi, Mayuko Sunohara, Masato Yoshihara, Atsushi Kunishima, Emiri Miyamoto, Hiroki Fujimoto, Kazuhisa Kitami, Kazumasa Mogi, Kaname Uno, Kosuke Yoshida, Satoshi Tamauchi, Akira Yokoi, Kaoru Niimi, Nobuhisa Yoshikawa, Ryo Emoto, Shigeyuki Matsui, Hiroaki Kajiyama

**Affiliations:** Department of Obstetrics and Gynecology, Nagoya University Graduate School of Medicine, 65 Tsurumai-cho, Showa-ku, Nagoya 466-8550, Japan; Institute for Advanced Research, Nagoya University, Furo-cho, Chikusa-ku, Nagoya 464-8601, Japan; Department of Obstetrics and Gynecology, Tosei General Hospital, 160 Nishioiwake-cho, Seto 489-8642, Japan; Department of Obstetrics and Gynecology, Nagoya University Graduate School of Medicine, 65 Tsurumai-cho, Showa-ku, Nagoya 466-8550, Japan; Department of Obstetrics and Gynecology, Nagoya University Graduate School of Medicine, 65 Tsurumai-cho, Showa-ku, Nagoya 466-8550, Japan; Department of Obstetrics and Gynecology, Nagoya University Graduate School of Medicine, 65 Tsurumai-cho, Showa-ku, Nagoya 466-8550, Japan; Department of Obstetrics and Gynecology, Nagoya University Graduate School of Medicine, 65 Tsurumai-cho, Showa-ku, Nagoya 466-8550, Japan; Discipline of Obstetrics and Gynaecology, Adelaide Medical School, Robinson Research Institute, University of Adelaide, Adelaide, SA 5000, Australia; Department of Obstetrics and Gynecology, Nagoya University Graduate School of Medicine, 65 Tsurumai-cho, Showa-ku, Nagoya 466-8550, Japan; Department of Gynecologic Oncology, Aichi Cancer Center, 1-1 Kanokoden, Chikusa-ku, Nagoya 464-8601, Japan; Department of Obstetrics and Gynecology, Nagoya University Graduate School of Medicine, 65 Tsurumai-cho, Showa-ku, Nagoya 466-8550, Japan; Department of Obstetrics and Gynecology, Nagoya University Graduate School of Medicine, 65 Tsurumai-cho, Showa-ku, Nagoya 466-8550, Japan; Division of Oncology, Department of Clinical Sciences, Lund University, Lund 221 84, Sweden; Department of Obstetrics and Gynecology, Nagoya University Graduate School of Medicine, 65 Tsurumai-cho, Showa-ku, Nagoya 466-8550, Japan; Department of Obstetrics and Gynecology, Nagoya University Graduate School of Medicine, 65 Tsurumai-cho, Showa-ku, Nagoya 466-8550, Japan; Department of Obstetrics and Gynecology, Nagoya University Graduate School of Medicine, 65 Tsurumai-cho, Showa-ku, Nagoya 466-8550, Japan; Department of Obstetrics and Gynecology, Nagoya University Graduate School of Medicine, 65 Tsurumai-cho, Showa-ku, Nagoya 466-8550, Japan; Department of Obstetrics and Gynecology, Nagoya University Graduate School of Medicine, 65 Tsurumai-cho, Showa-ku, Nagoya 466-8550, Japan; Department of Biostatistics, Nagoya University Graduate School of Medicine, 65 Tsurumai-cho, Showa-ku, Nagoya 466-8550, Japan; Department of Biostatistics, Nagoya University Graduate School of Medicine, 65 Tsurumai-cho, Showa-ku, Nagoya 466-8550, Japan; Department of Obstetrics and Gynecology, Nagoya University Graduate School of Medicine, 65 Tsurumai-cho, Showa-ku, Nagoya 466-8550, Japan

**Keywords:** ovarian cancer, ascites cytology, micro-metastasis, R0 surgery

## Abstract

**Background:**

In ovarian cancer (OvCa), achieving complete resection (RO) in initial surgery is crucial for improving prognosis. However, patients with undetected microscopic metastasis post-RO surgery often have poorer outcomes. This study explores prognostic factors for OvCa patients who underwent RO surgery, focusing on the role of ascites cytology as an indicator of microscopic peritoneal metastasis.

**Methods:**

We analyzed data from 975 OvCa cases in the Tokai Ovarian Tumor Study Group database (1986–2019). Excluding patients without chemotherapy or with distant metastasis, we examined prognostic factors using Cox regression analysis. Propensity score (PS) methods balanced the cytology-positive and -negative groups, with subgroup analysis for clinical stage and ascites volume.

**Results:**

Multivariate analysis identified FIGO stage III and positive ascites cytology as poor prognostic factors for overall and progression-free survival. After PS adjustment, positive ascites cytology also shortened progression-free intervals post-recurrence, especially in cases with peritoneal or lymph node metastasis. Subgroup analysis revealed a more substantial prognostic impact of positive ascites cytology in early-stage cases.

**Conclusion:**

The present results suggest that in OvCa patients with the R0 status, the presence of tumor cells in ascites is an independent negative prognostic factor and may be an indicator of peritoneal micro-metastasis.

## Teaser

Our study suggested that, in ovarian cancer patients with the R0 status, positive ascites cytology is an independent negative prognostic factor and may be an indicator of peritoneal micro-metastasis.

## Introduction

Ovarian cancer (OvCa) is only diagnosed in the advanced stages [1] and most patients present with fluid accumulation in the peritoneal cavity, called ascites [2]. Tumor debulking surgery, also known as cytoreductive surgery, plays a pivotal role in the management of OvCa [3]. Its primary aim is to remove as much tumor mass as possible, reflecting the principle that fewer residual cancer cells remaining post-surgery may increase the response rate to subsequent chemotherapy and reduce the development of chemoresistance [4]. Surgical procedures that result in no visible residual disease are referred to as R0 surgery [5–7], and the R0 status has been consistently associated with better survival outcomes in OvCa patients [4,7–11]. Therefore, achieving the R0 status in the initial surgery is the ultimate goal in the early phase of OvCa management [8].

Recent advances in OvCa treatment, including the use of neoadjuvant chemotherapy before surgery [12,13], have led to higher rates of complete cytoreduction, namely, the R0 status [6]. Under these conditions, further investigations of prognostic factors in R0 cases are required. Furthermore, whether the R0 status is achieved is based, from a biological aspect, on macroscopic observations during surgery and thus, the presence and effects of microscopic metastasis have been the focus of research [14,15]. Ascites cytology is assumed to possess significant information on peritoneal micro-metastasis [16,17], and we previously identified a positive ascites cytology as a strong independent prognostic factor [18]. However, its impact on R0 cases was not investigated in detail. Limited information is currently available on the comprehensive long-term clinical significance of ascites cytology in R0 cases, mainly due to the small number of analyzable patients.

Therefore, we herein examined the long-term impact of ascites cytology on the prognosis of epithelial OvCa cases in which R0 resection was achieved in the initial surgery with a multi-institutional large-scale clinical cohort. To precisely estimate its prognostic significance, adjustments by the propensity score (PS) weighting method were applied. Detailed subgroup analyses, including stratification by clinical stages and histotypes, were also performed.

## Patients and methods

### Study participants

This multi-institutional retrospective cohort study utilized clinical data from the medical records of patients registered with the Tokai Ovarian Tumor Study Group between January 1986 and September 2019. Only cases that achieved the R0 status during the initial surgery were included. Patients who did not receive chemotherapy nor had distant metastasis (stage IV) were excluded. The resulting study cohort was retrospectively analyzed. Ethical approval was obtained from the Ethics Committee of Nagoya University (No. 2006–0357), and the study was conducted in accordance with the principles of the Declaration of Helsinki.

### Surgery, chemotherapy, and follow-up

Primary debulking surgery was performed, consisting of total hysterectomy, bilateral salpingo-oophorectomy, ascites cytology, biopsy, omentectomy, staging lymphadenectomy, and a peritoneal evaluation [19]. R0 resection of the tumor was achieved in all cases. Due to the inclusion of patients across several decades, chemotherapy regimens varied; detailed descriptions of adjuvant chemotherapy protocols for each period were provided in our previous study [20]. Follow-ups included regular pelvic examinations using ultrasonography, enhanced computed tomography scans, and tumor marker evaluations. Magnetic resonance imaging or positron emission tomography was utilized as needed. Recurrence was clinically defined as the development of ascites, a detectable mass, or elevated tumor markers according to Gynecologic Cancer InterGroup criteria [21]. Progression-free survival (PFS) was defined as the time from the initial surgery to the last follow-up or tumor recurrence. Post-recurrence survival (PRS) was calculated by subtracting PFS from overall survival (OS) in cases of recurrence. PFS for patients with recurrence was also referred to as the progression-free interval (PFI).

### Statistical analysis

Statistical analyses were conducted using R statistical software (version 4.2.3) and RStudio (version 2023.06.0 + 421). The PS method was employed to adjust for imbalances between the two groups, with scores calculated by a logistic regression model using the following covariates: disease stage (FIGO I/II vs. III), histological type (serous vs. others), CA-125 levels (logged), the performance of complete surgical staging, and the volume of ascites. Sensitivity analysis confirmed that the selection of variables was reasonable. Adjustments of the study cohorts were conducted using the inverse probability weighting of treatment approach, where each individual was weighted by the inverse probability of a positive or negative cytology. Patient background before and after adjustment was summarized in Table S1. Statistical comparisons between the two groups were performed using the student’s *t*-test for continuous variables and the chi-squared or Fisher’s exact test for categorical variables. Survival differences between the groups were visualized using the Kaplan–Meier method on the adjusted cohort and assessed by the log-rank test. In subgroup analyses, the estimation of the hazard ratio (HR) of ascites cytology was performed by stratifying each variable, and conducting multivariate Cox’s regression (variables; age, FIGO staging, histotype, logged CA-125, ascites volume, Complete staging surgery, and ascites cytology. The variable used in stratification was dropped). The significance of differences was assessed as two-sided with a *P* value < .05.

## Results

### Baseline characteristics of patients

Among 5268 patients with malignant ovarian tumors, 975 that met the inclusion criteria were selected (Fig. S1). Ascites cytology was positive in 353 patients (36.2%) and negative in 622 (63.8%). Baseline patient characteristics are summarized in Table 1. As expected, the number of stage I cases was higher than that of stage II/III. The serous histotype had a significantly higher positive cytology rate, reflecting its higher likelihood to develop peritoneal dissemination. The most abundant histotype was clear-cell carcinoma, which is more prevalent in East Asia than in Western countries and is often diagnosed at an earlier stage than the more frequent serous histotype. Patients with a positive ascites cytology retained a higher ascites volume at the time of the initial surgery, indicating that the volume and cytology of ascites both reflected disease progression.

**Table 1:** Baseline characteristics of patient in low and high ascites volume classes

	Ascites cytology		
	Negative	Positive	*P*-value
*n*	622	353	
Age, years (SD)	53.57 (10.64)	54.64 (11.26)	.141
Tumor stage, *n* (%)			
I	423 (68.0)	163 (46.2)	<.001
II	106 (17.0)	61 (17.3)	.995
III	93 (15.0)	129 (36.5)	<.001
Histotype, *n* (%)			
Serous	115 (18.5)	128 (36.3)	<.001
Clear-cell	258 (41.5)	131 (37.1)	.204
Mucinous	76 (12.2)	25 (7.1)	.016
Endometrioid	152 (24.4)	61 (17.3)	.012
Others	21 (3.4)	8 (2.3)	.433
Tumor markers, IU/ml (SD)			
CA125	782.00 (4077.48)	864.19 (1889.41)	^a^.721
CA19-9	2688.04 (29428.73)	713.64 (4152.65)	^a^.229
CA72-4	39.33 (143.81)	71.11 (237.29)	^a^04
CEA	6.47 (29.08)	8.40 (38.76)	^a^.48
Complete staging surgery, *n* (%)	320 (51.4)	198 (56.1)	.184
Ascites volume ≥500 ml, *n* (%)	49 (7.9)	58 (16.4)	<.001

Data are presented as mean ± standard deviation or proportion (%).

Student’s *t*-test, chi-square test, or Fisher’s exact test was used as appropriate.

SD, standard deviation. CA, cancer antigen.

^a^Logarithmically transformed when analyzed.

### Effects of a positive ascites cytology in R0 cases

**Figure 1:**
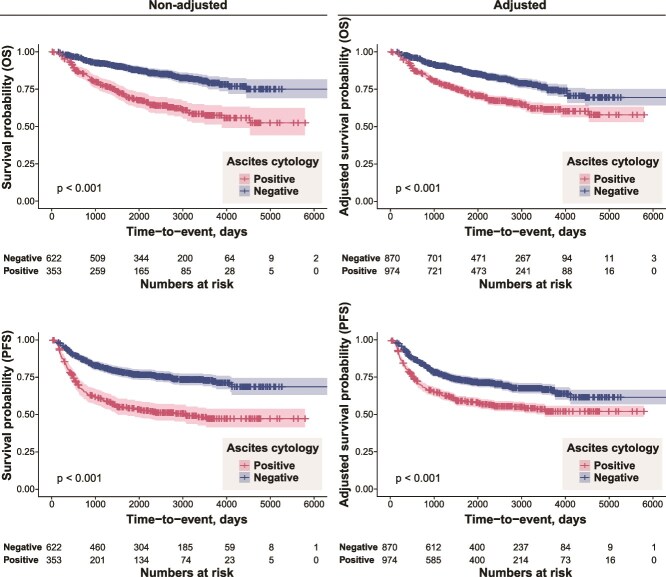
(A-B) Kaplan–Meier curves of OS stratified by ascites cytology results in the initial surgery with (A) unadjusted and (B) adjusted cohorts. (C-D) Kaplan–Meier curves of PFS stratified by ascites cytology results in the initial surgery with (C) unadjusted and (D) adjusted cohorts.

To estimate the long-term prognostic impact of a positive ascites cytology, we initially conducted uni- and multivariate Cox regression analyses of the survival outcomes of patients (Table 2). Among the clinical factors included, a positive ascites cytology was associated with a poor prognosis [HR for PFS, 1.72, 95% confidence interval (CI) 1.37–2.18, *P* < .001; HR for OS, 1.96, 95% CI 1.48–2.61, *P* < .001]. We then adjusted for differences between the ascites-positive and -negative groups using the PS-based approach because there was an imbalance in patient backgrounds. PS scores were calculated using a logistic regression model with relevant clinical factors, including disease stage (FIGOI/II vs. III), histological type (serous vs. others), CA-125 levels (logged), the performance of complete surgical staging, and the volume of ascites. Kaplan–Meier curves of OS and PFS were depicted with datasets before and after adjustments (Fig. 1A–D), and the results obtained indicated that a positive ascites cytology had a significant negative impact on both OS and PFS in OvCa patients who achieved the R0 status in the initial surgery.

**Table 2:** Uni and multivariate analysis

	PFS				OS			
	Univariate		Multivariate		Univariate		Multivariate	
	HR (95% CI)	*P*-value	HR (95% CI)	*P*-value	HR (95% CI)	*P*-value	HR (95% CI)	*P*-value
FIGO stage								
I	0.35 (0.28–0.44)	< .001	ref	ref	0.31 (0.24–0.42)	<.001	ref	ref
II	0.98 (0.73–1.31)	0.881	1.51 (1.09–2.10)	.0134	1.25 (0.90–1.73)	.190	2.19 (1.50–3.20)	<.001
III	3.62 (2.90–4.53)	<.001	3.16 (2.37–4.22)	<.001	3.28 (2.51–4.29)	<.001	3.47 (2.44–4.94)	<.001
Histotype								
Serous	2.14 (1.71–2.69)	<.001	ref	ref	1.77 (1.34–2.35)	<.001	ref	ref
Clear-cell	0.89 (0.71–1.12)	.332	1.12 (0.83–1.50)	.461	0.96 (0.73–1.26)	.745	1.25 (0.87–1.78)	.222
Mucinous	0.71 (0.48–1.06)	.0966	1.02 (0.64–1.62)	.945	0.97 (0.63–1.50)	.908	1.49 (0.89–2.50)	.132
Endometrioid	0.54 (0.40–0.75)	<.001	0.72 (0.50–1.05)	.0888	0.48 (0.32–0.72)	<.001	0.70 (0.43–1.12)	.138
Others	0.69 (0.33–1.46)	.333	0.61 (0.28–1.30)	.199	1.01 (0.48–2.15)	.974	1.00 (0.46–2.17)	.992
Age	1.02 (1.01–1.03)	.00107	1.01 (1.00–1.02)	.0885	1.01 (1.00–1.03)	.0229	1.01 (0.99–1.02)	.302
CA125	1.19 (1.12–1.26)	<.001	1.06 (0.99–1.14)	.113	1.14 (1.06–1.22)	<.001	0.99 (0.91–1.09)	.868
Complete staging surgery^a^	0.83 (0.67–1.04)	.108	0.78 (0.62–0.97)	.0273	0.89 (0.68–1.16)	.392	0.87 (0.66–1.14)	.322
Ascites >500 ml^b^	1.53 (1.12–2.07)	.00712	1.21 (0.88–1.66)	.246	1.83 (1.30–2.59)	<.001	1.60 (1.11–2.31)	.0115
Ascites cytology^b^	2.38 (1.91–2.97)	<.001	1.72 (1.37–2.18)	<.001	2.64 (2.02–3.46)	<.001	1.96 (1.48–2.61)	<.001

^a^Complete staging surgery: total hysterectomy, bilateral salpingo-oophorectomy, ascites cytology, biopsy, omentectomy, staging lymphadenectomy, and a peritoneal evaluation.

^b^Volume and cytology of ascites were evaluated upon initial cytoreductive surgery.

### PFI and PRS

**Figure 2:**
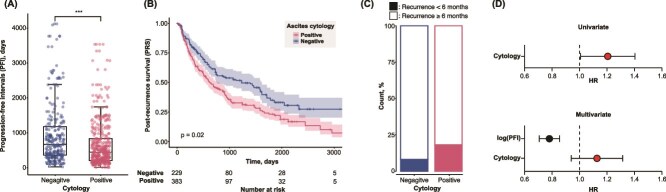
(A) Progression-free intervals (PFI) of recurrent cases in positive and negative ascites cytology groups. (B) Kaplan–Meier curves of PRS stratified by ascites cytology results. (C) Bar graph showing the rate of recurrence within 6 months against all recurrent cases in positive and negative ascites cytology groups. (D) HR values of ascites cytology in univariate and bivariate (covariate: Logged PFI value) cox regression analyses.

PFI and PRS were analyzed with the PS-based pseudo-population dataset (Fig. 2A). When patients with recurrence after R0 surgery were filtered, PFI was significantly shorter in cases with a positive ascites cytology than in those with a negative cytology (*P* < .001). Cytology results in the initial surgery were also associated with shorter PRS (Fig. 2B), indicating that cytology results affected the condition of the disease even after the event of recurrence. The incidence of recurrence within 6 months was higher in the cytology-positive group (Fig. 2C). In Fig. 2D, HR of a positive ascites cytology for PRS was calculated as 1.195 (95% CI: 1.014–1.409). When PFI was introduced as a covariable in the multivariate analysis, HR of ascites cytology for PRS became 1.117 (95% CI: 0.945–1.320), while that of PFI (logged) was 0.779 (95% CI: 0.708–0.856), indicating that a positive cytology was associated with a poorer prognosis in OvCa patients with the R0 status by shortening PFI after the initial cytoreductive surgery.

### Site of recurrence in patients with and without a positive ascites cytology

**Figure 3:**
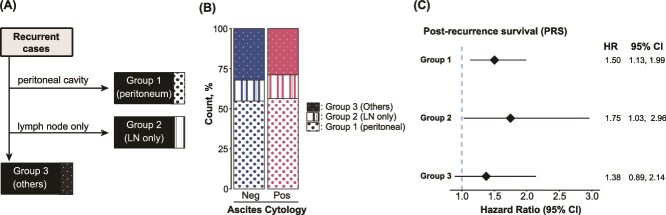
(A) Flow diagram showing the sorting of recurrent cases into three site of recurrence groups. (B) the rate of recurrence groups within recurrent cases in positive and negative ascites cytology groups. (C) the effects of ascites cytology on PRS after stratification by Kaplan–Meier curves of PRS stratified by site of recurrence groups.

Based on the site of recurrence, patients with recurrence were classified into three groups as shown in Fig. 3A: Group 1 (peritoneum), Group 2 (LN alone), and Group 3 (others). In Pearson’s chi-squared test, no significant difference was observed in the distribution of recurrence sites between the ascites-positive and -negative groups (*P* = .199, Fig. 3B). HR of a positive ascites cytology for PRS was assessed in each group and compared. As expected, among patients in Group 1 with recurrence in the peritoneal cavity, a positive ascites cytology had a negative impact on PRS (HR: 1.50, 95% CI: 1.13–1.99). A negative impact was also confirmed for patients in Group 2 with recurrence at LN only (HR: 1.75, 95% CI: 1.03–2.96), but not for patients in Group 3 (HR: 1.38, 95% CI: 0.89–2.14). These results suggest that a positive ascites cytology increased the risk of cases with intra-peritoneal or LN recurrence.

### Subgroup analysis

**Figure 4:**
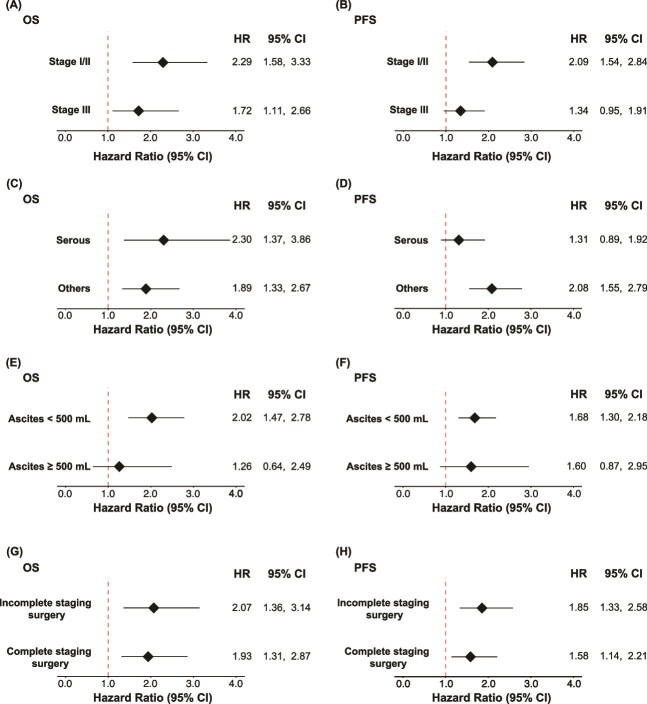
(A-D) Results of subgroup analyses of (A-B) FIGO staging, (C-D) histology, (E-F) accumulated volume of ascites, and (G-H) the status of complete staging surgery groups depicted as HR values of positive ascites cytology.

**Figure 5:**
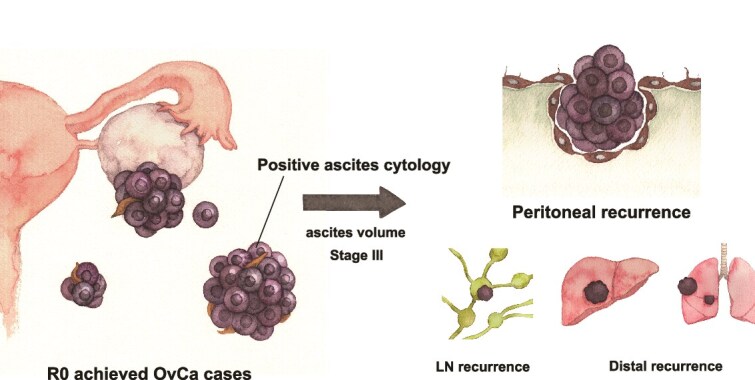
Schematic image based on the present results.

We then performed subgroup analyses and evaluated the relative HR of a positive ascites cytology for OS and PFS in each subgroup based on the disease stages, histotypes, accumulated ascites volume, and the status of full staging surgery. In the examination with FIGO stages, a positive ascites cytology had a greater negative impact on both OS and PFS at an earlier stage (Fig. 4A-B). In the analysis based on histotypes, ascites cytology results had a negative impact on both serous and non-serous histologies, especially for OS (Fig. 4C-D). Regarding intra-abdominal fluid accumulation, ascites cytology had a greater negative impact in the smaller ascites retention group (Fig. 4E-F). As for patients with ascites >500 ml, the 95% CI for HR of ascites cytology crossed one in both OS and PFS, indicating that the outcome of cases with higher ascites accumulation but negative cytology did not differ much when compared with positive cytology cases. The status of full staging surgery did not exert marked effects, while cytology results had a significant negative impact in both the complete and incomplete staging surgery groups; however, HR increased in cases in which complete staging surgery was not achievable (Fig. 4G-H). Obtained results were summarized in Fig. 5 as a schematic image.

## Discussion

A positive ascites cytology in the primary or interval debulking surgery has been identified as an independent negative prognostic factor. Furthermore, positive ascites cytology results in stage I epithelial OvCa leads to a diagnosis of stage IC3 [16]. In OvCa cases, the R0 resection of a tumor in debulking surgery is a positive prognostic indicator [7,9–11]. However, whether a tumor is fully resected is based on a macroscopic examination performed during surgery. Among patients who undergo R0 resection, a prognostic subclassification based on the level of micro-metastasis has been demanded. It currently remains unclear whether ascites cytology is a significant predictor of the outcomes of R0 cases in a long follow-up period. In the present study, we enabled this examination by utilizing multi-institutional cohort data from 1986 with PS-based adjustments and revealed that a positive ascites cytology was an independent prognostic factor among R0 cases.

The main result of the present study was that ascites cytology was associated with a poorer prognosis by shortening the recurrence-free interval. When a Kaplan–Meier curve of PRS was depicted with stratification by cytology results in the initial surgery, a positive ascites cytology was associated with shorter PRS. When the HR of positive cytology results was calculated for PRS in the univariate Cox regression, a negative impact was noted. Furthermore, following the introduction of logged PFI values as a covariable, the greater impact of PFI on PRS was highlighted and the negative impact of a positive cytology was diminished. Therefore, the negative prognostic impact of a positive ascites cytology on PRS by shortening PFI was highlighted.

Due to its multi-institutional, large-scale design, subgroup analyses were possible in this study and revealed that the negative impact of ascites cytology results varied according to the disease stage, even among R0 cases. Notably, the impact of a positive ascites cytology decreased as the disease stage increased, reinforcing the importance of separating stage IC3 in early-stage OvCa. In the same manner, ascites cytology had a greater negative impact on cases with the accumulation of a smaller volume of ascites. Therefore, the importance of peritoneal washing cytology was reconfirmed. Biologically, OvCa cells are considered to be present in ascites as a form of multicellular spheroid to avoid anoikis [22]. With support from co-existing cells, OvCa cells appear to attach to the peritoneal wall more easily and proliferate more rapidly at a metastatic lesion [22]. Since OvCa cells detected in cytology testing are often captured as cell aggregates, the existence of pathologically identified cancer cells in ascites may reflect the degree of cellular-level peritoneal micro-metastasis in R0 cases. Therefore, cytology results are considered to be helpful in subgrouping the high-risk group for disease recurrence. This speculation is supported by the results obtained in this multi-institutional cohort with a long-term follow-up.

As strength of the present study, a central pathological review system was adopted and clinical information from affiliated institutions was analyzed in a uniform manner. Since all the institutions involved were affiliated hospitals of Nagoya University, the surgical procedures and administration of chemotherapy were relatively uniform and consistent. On the other hand, the design of this study with a long follow-up period to gain an adequate cohort size may be one of its limitations because surgical and medical homogeneities may be a tradeoff. Furthermore, there may have been some confounding factors, including the effects of medical comorbidities, because of the lack of data obtained on comorbidities in a uniform manner. Most of these limitations were derived from the retrospective design of the present study and, thus, further studies are needed to verify the results obtained.

## Conclusion

We herein identified a positive ascites cytology as a poor prognostic factor for OvCa, even in R0 cases. A positive ascites cytology appeared to be associated with the prognosis of patients with recurrence. Positive results need to be interpreted as an indicator of cellular-level micro-metastasis in R0 cases, and further attention is needed during the post-surgical management of OvCa when ascites cytology is positive.

## Supplementary Material

supplementary_material_R1_hyaf046

STROBE_checklist_cohort_hyaf046
