# Supplementary material for: Long-term prognostic significance of ascites cytology in ovarian cancer cases in which R0 resection was achieved in the initial surgery: a multi-institutional retrospective cohort study
Source: Jpn J Clin Oncol. 2025 Mar 20;55(7):743–50. doi: 10.1093/jjco/hyaf046 (PMC12230295; doi:10.1093/jjco/hyaf046)
Supplement: supplementary_material_R1_hyaf046 [file supplementary_material_r1_hyaf046.docx]

**SUPPLEMENTARY MATERIAL**

**Long-term prognostic significance of ascites cytology in ovarian cancer cases in which R0 resection was achieved in the initial surgery: A multi-institutional retrospective cohort study**

Shohei Iyoshi^1,2#^, Mayuko Sunohara^3,#^, Masato Yoshihara^1,*^, Atsushi Kunishima^1^, Emiri Miyamoto^1^, Hiroki Fujimoto^1,4^, Kazuhisa Kitami^1,5^, Kazumasa Mogi^1^, Kaname Uno^1,6^, Kosuke Yoshida^1^, Satoshi Tamauchi, Akira Yokoi^1^, Kaoru Niimi^1^, Nobuhisa Yoshikawa^1^, Ryo Emoto^7^, Shigeyuki Matsui^7^, Hiroaki Kajiyama^1^

^1^ Department of Obstetrics and Gynecology, Nagoya University Graduate School of Medicine, 65 Tsurumai-cho, Showa-ku, Nagoya, Japan

^2^ Institute for Advanced Research, Nagoya University, Furo-cho, Chikusa-ku, Nagoya, Japan

^3^ Department of Obstetrics and Gynecology, Tosei General Hospital, 160 Nishioiwake-cho, Seto 489-8642, Japan

^4^ Discipline of Obstetrics and Gynaecology, Adelaide Medical School, Robinson Research Institute, University of Adelaide, Adelaide, SA, Australia

^5^ Department of Gynecologic Oncology, Aichi Cancer Center, 1-1 Kanokoden, Chikusa-ku, Nagoya, Japan

^6^ Division of Oncology, Department of Clinical Sciences, Lund University, Lund, Sweden

^7^ Department of Biostatistics, Nagoya University Graduate School of Medicine

^#^These authors contributed equally to this work.

*Corresponding Author

**Contact information:**

Masato Yoshihara

Department of Obstetrics and Gynecology, Nagoya University Graduate School of Medicine

Showa-ku, Nagoya, Japan

TEL.: +81-52-744-2262

FAX: +81-52-744-2268

E-mail: myoshihara1209@med.nagoya-u.ac.jp

**Contents**

1. Supplementary Figure
2. Supplementary Table

**Figure S1.** Flow chart of the study subjects.


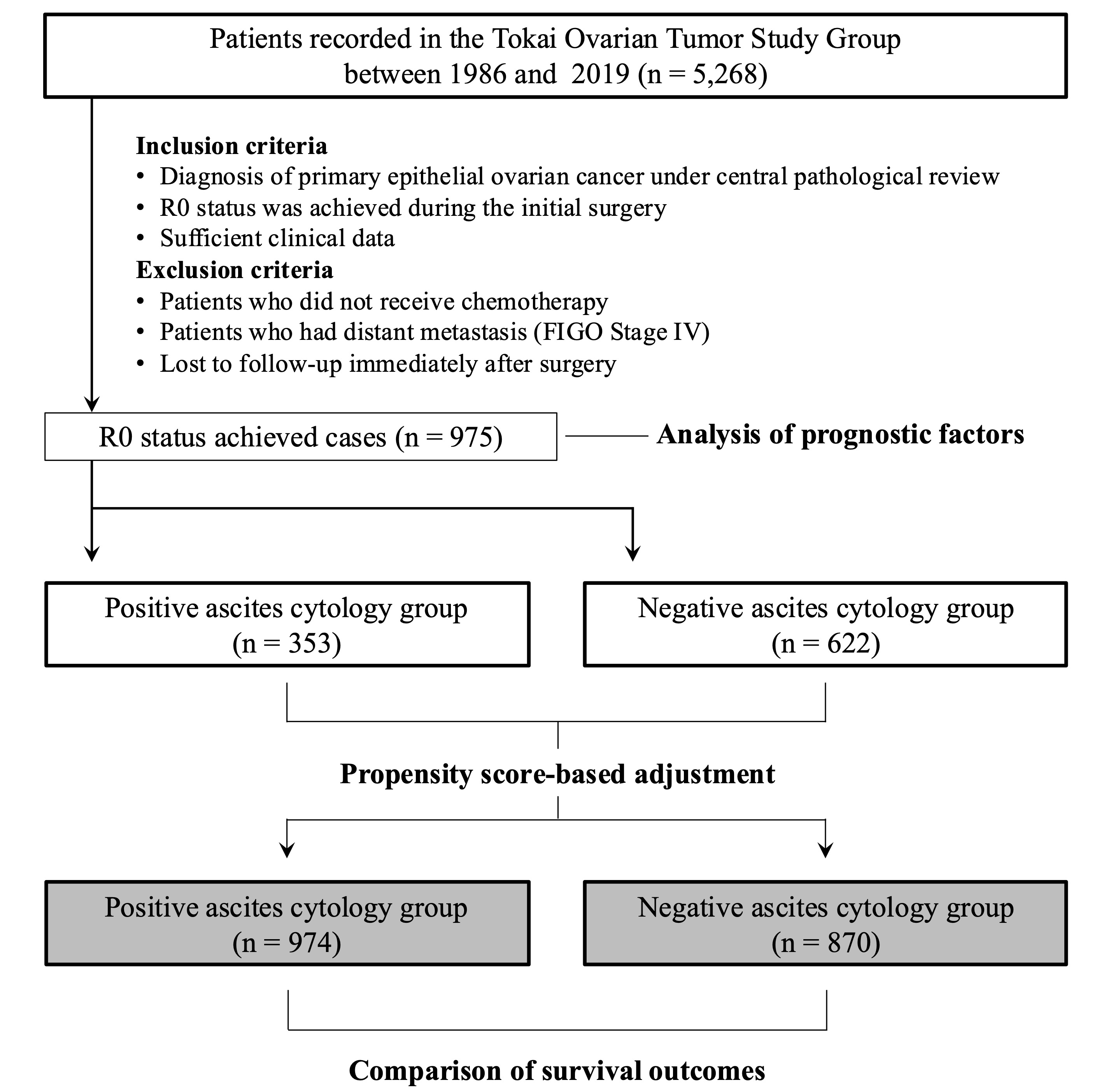


**Table S1.** Patient backgrounds before and after IPTW adjustment
